# Inhibition of Renal Tubular Epithelial Mesenchymal Transition and Endoplasmic Reticulum Stress-Induced Apoptosis with Shenkang Injection Attenuates Diabetic Tubulopathy

**DOI:** 10.3389/fphar.2021.662706

**Published:** 2021-08-02

**Authors:** Wen-Wen Wang, Ying-Lu Liu, Mei-Zi Wang, Huan Li, Bu-Hui Liu, Yue Tu, Can-Can Yuan, Qi-Jun Fang, Jia-Xin Chen, Jie Wang, Yan Fu, Zi-Yue Wan, Yi-Gang Wan, Wei Wu

**Affiliations:** ^1^ Department of Traditional Chinese Medicine, Nanjing Drum Tower Hospital Clinical College of Nanjing University of Chinese Medicine, Nanjing, China; ^2^ Department of Nephrology, Wenzhou Hospital of Integrated Traditional Chinese and Western Medicine, Wenzhou, China; ^3^ Department of Traditional Chinese Medicine, Nanjing Drum Tower Hospital, The Affiliated Hospital of Nanjing University Medical School, Nanjing, China; ^4^ Nephrology Division, Affiliated Hospital of Nanjing University of Chinese Medicine, Nanjing, China; ^5^ Department of Traditional Chinese Medicine Health Preservation, Acupuncture, Moxibustion and Massage College, Health Preservation and Rehabilitation College, Nanjing University of Chinese Medicine, Nanjing, China; ^6^ Graduate School of Social Sciences, Faculty of Social Sciences, Hitotsubashi University, Tokyo, Japan

**Keywords:** diabetic tubulopathy, shenkang injection, apoptosis, renal tubular epithelial mesenchymal transition, endoplasmic reticulum stress

## Abstract

**Background:** The proximal renal tubule plays a critical role in diabetic kidney disease (DKD) progression. Early glomerular disease in DKD triggers a cascade of injuries resulting in renal tubulointerstitial disease. These pathophysiological responses are collectively described as diabetic tubulopathy (DT). Thus, therapeutic strategies targeting DT hold significant promise for early DKD treatment. Shenkang injection (SKI) has been widely used to treat renal tubulointerstitial fibrosis in patients with chronic kidney disease in China. However, it is still unknown whether SKI can alleviate DT. We designed a series of experiments to investigate the beneficial effects of SKI in DT and the mechanisms that are responsible for its effect on epithelial-to-mesenchymal transition (EMT) and endoplasmic reticulum (ER) stress-induced apoptosis in DT.

**Methods:** The modified DKD rat models were induced by uni-nephrectomy, streptozotocin intraperitoneal injection, and a high-fat diet. Following the induction of renal injury, these animals received either SKI, rosiglitazone (ROS), or vehicle, for 42 days. For *in vitro* research, we exposed NRK-52E cells to high glucose (HG) and 4-phenylbutyric acid (4-PBA) with or without SKI or ROS. Changes in parameters related to renal tubular injury and EMT were analyzed *in vivo.* Changes in the proportion of apoptotic renal tubular cells and ER stress, and the signaling pathways involved in these changes, were analyzed both *in vivo* and *in vitro*.

**Results:** SKI and ROS improved the general condition, the renal morphological appearance and the key biochemical parameters, and attenuated renal injury and EMT in the rat model of DKD. In addition, SKI and ROS alleviated apoptosis, inhibited ER stress, and suppressed PERK-eIF2α-ATF4-CHOP signaling pathway activation both *in vivo* and *in vitro*. Notably, our data showed that the regulatory *in vitro* effects of SKI on PERK-eIF2α-ATF4-CHOP signaling were similar to those of 4-PBA, a specific inhibitor of ER stress.

**Conclusion:** This study confirmed that SKI can alleviate DT in a similar manner as ROS, and SKI achieves this effect by inhibiting EMT and ER stress-induced apoptosis. Our findings thereby provide novel information relating to the clinical value of SKI in the treatment of DT.

## Introduction

As one of the major microvascular complications of diabetes mellitus (DM), diabetic kidney disease (DKD) has become a common health problem in China (Ma, 2018). Research has shown that 30–40% of patients with DM eventually develop DKD and that this is a dominant cause of end-stage renal disease (Umanath and Lewis, 2018). Although there is now extensive knowledge regarding renal damage in DKD, the pathogenesis of DKD progression remains controversial, especially for patients with early DKD who do not exhibit mass proteinuria (Alicic et al., 2017; Chen et al., 2017). It has been reported that early glomerular disease in DKD triggers a cascade of injuries that result in renal tubulointerstitial disease. An increasing body of *in vivo* and *in vitro* evidence now indicates that the proximal renal tubule plays a critical role in early DKD (Vallon, 2011). This has led to the term diabetic tubulopathy (DT) (Tang et al., 2011). The concept of DT mainly stems from the ability of the proximal tubular epithelial cells (PTECs) to secrete proinflammatory and profibrotic factors and to undergo epithelial mesenchymal transition (EMT) upon stimulation by various metabolic substrates of DM (Zeni et al., 2017). Accordingly, a variety of factors have been detected that have the ability to cause injury to the renal tubules, including interleukin-6 (IL-6), transforming growth factor-β (TGF-β), vascular endothelial growth factor (VEGF) and till-like receptor 4 (TLR4); several novel pathogenic mechanisms have also been demonstrated in PTECs, including mitochondrial fragmentation, autophagy, and apoptosis (Tang et al., 2010; Alicic et al., 2017; Lee et al., 2019). Consequently, it is highly evident that therapeutic strategies that target DT hold significant promise for patients with early DKD.

Lee et al. (2019) reported that EMT of PTECs is one of the major pathogenic events of DT; this process involves a series of events in which PTECs not only lose several epithelial characteristics including E-cadherin but also acquire typical properties of mesenchymal cells, such as the expression of collagen type I (collagen I), vimentin, and α-smooth muscle actin (α-SMA). In addition, the damage incurred by PTECs during DKD is known to result in the accumulation of unfolded proteins in the endoplasmic reticulum (ER), thus causing a severe unfolded protein response (UPR). Persistent UPR can eventually lead to cellular apoptosis (Cunard, 2015; Cybulsky, 2017). Therefore, if we are to ameliorate DT, it is vital that we inhibit EMT and ER stress-induced apoptosis in PTECs.

It is well known that three membrane proteins in the ER can function as stress sensors: double-stranded RNA (PKR)-activated protein kinase-like eukaryotic translation initiation factor 2α (eIF2α) kinase (PERK), inositol-requiring transmembrane kinase/endoribonuclease 1, and activating transcription factor 6. Normally, these proteins bind to chaperone glucose-regulated protein 78 (GRP78) to form an inactive protein complex. However, under stressful conditions, these protein sensors can be released from the protein complexes to initiate ER stress; this occurs when misfolded proteins accumulate and causes the sequestration of GRP78. PERK, as the ER stress sensor that can phosphorylate eIF2α, then causes the reduction of protein synthesis and the upregulation of ER stress-related protein expression, including activating transcription factor 4 (ATF4) and C/EBP homologous protein (CHOP). This signaling pathway eventually results in the restoration of cellular homeostasis, particularly with regards to protein translation and folding. The activation of eIF2α can selectively promote protein translation, thus playing a vital role in ER stress-induced apoptosis. The family of caspase12 and Bcl-2 proteins also play an important role in this signaling pathway (Kitamura, 2008a; Kitamura, 2009; Oakes and Papa, 2015; Oakes, 2020).

Shenkang injection (SKI) has been approved by the National Medical Products Administration for the treatment of chronic kidney disease (CKD) as a modern preparation of Chinese patent medicine (Z20040110) and has been widely used in China to treat renal tubulointerstitial fibrosis (TIF) in patients with CKD for almost 20 years (Song et al., 2019; Zou et al., 2020). In a previous study, Xu et al. reported that SKI, and its major active component emodin, can inhibit the proliferation and apoptosis of mesangial cells induced by high glucose (HG) conditions (Xu et al., 2016). More recently, we also demonstrated that SKI can attenuate TIF *in vivo* by blocking pericyte-myofibroblast transition in the kidneys of rats with obstructive nephropathy (Liu et al., 2019). However, to the best of our knowledge, it is still unknown whether SKI can alleviate DT.

In this study, we used SKI as a new therapeutic strategy to improve EMT and ER stress-induced apoptosis in DT. We compared the effects of SKI to rosiglitazone (ROS), a conventional insulin sensitizing agent that is used in the clinic. In addition to this, we comprehensively evaluated the mechanisms that underlie the beneficial effects of SKI on diabetic kidneys and PTECs, paying particular attention to the ER stress-related PERK-eIF2α-ATF4-CHOP signaling pathway.

## Materials and Methods

### Preparation and Quality Control of SKI

SKI was purchased from Xi’an Century Shenkang Pharmaceutical Industry Co., Ltd. (Xi’an, China) and consisted of extracts from a defined mixture of Chinese herbs: radix et rhizoma rhei (*Rheum palmatum* L., Dahuang), radix astragali [*Astragalus membranaceus* (Fisch.) Beg. Huangqi], radix salviae miltiorrhizae (*Salvia miltiorrhiza* Bunge., Danshen), and Flos carthami (*Carthamus tinctorius* L., Honghua). One injection of SKI (20 ml) contains 6 g of extracts in total. The extraction method and the production process for SKI is protected by patent law in China and are both subjected to strict quality control; the main components are also subjected to standardization. The batch number of SKI used in this study was 201,607,021. The quality of the SKI was determined by fingerprint analysis and high performance liquid chromatography (HPLC), as described previously by Yao et al. (2015) and Xu et al. (2017). The main bioactive components of anthraquinones, including *Aloe-emodin* (C_15_H_10_O_5_; CAS: 481-72-1), *Rhein* (C_15_H_8_O_6_; CAS: 478-43-3), *Emodin* (C_15_H_10_O_5_; CAS: 518-82-1), *Chrysophanol* (C_15_H_10_O_4_; CAS: 481-74-3), and *Physcion* (C_16_H_12_O_5_; CAS: 521-61-9) (shown in Supplemental Figure S1), exhibited high levels of stability in the products of SKI purchased in different places and used in this study.

### Animals and Treatments

Forty-eight male Sprague Dawley rats, weighing 200 ± 10 g, were provided by the Animal Center of Nanjing Medical University (Nanjing, China). All animal handling and experimental procedures were performed in accordance with local ethics committees and the National Institutes of Health Guide for the Care and Use of Laboratory Animals. All efforts were made to minimize animal suffering and reduce the number of animals used. All procedures involving animals were approved by the Animal Ethics Committee of Nanjing University Medical School (Qualified number: SYXK 2014-0052). The feeding conditions of rats were described previously (Han et al., 2019). The 48 rats were divided into four groups using a random number table, with 5, 14, 14, and 15 rats in the Sham, Vehicle, SKI, and ROS groups, respectively. For rats in the Sham group, the left kidney was exposed during surgery; the rats were then given distilled water and a standard diet for 20 weeks until finished. In contrast, the rats in the other three groups were given a 40% high-fat diet containing 19.8% fat, 22.3% crude protein, and 44.6% carbohydrates, for 4 weeks. The rats in these three groups were then subjected to left nephrectomy and received two intraperitoneal injections (on the right side of their bodies; 3 days apart) of streptozocin (STZ) at a dosage of 35 mg/kg. This process last for 10 weeks and finally established a modified rat model of DKD, as described in detail in our previous studies (Mao et al., 2015; Wu et al., 2017; Wu et al., 2018; Han et al., 2019). Once the model had been established, we then used gastric gavage to administer appropriate daily treatments: SKI was given to rats in the SKI group, while rats in the Vehicle and ROS groups were treated with 2 ml of distilled water or ROS, respectively. After 6 weeks of treatment with the different interventions, we collected urine firstly, and then anesthetized rats and collected blood, finally sacrificed all rats by cardiac puncture at the same time point. Kidneys were collected for the detection of various indicators; the experimental process is depicted in Supplemental Figure S2. In the clinic, 100 ml/day of SKI is normally used to treat a 60 kg non-dialysis patient with CKD. Based on standard animal conversion formulae, the effective amount of SKI in a rat weighing 200 g was determined to be equivalent to 5 g/kg/day. ROS was purchased from GlaxoSmithKline Co. (United Kingdom); the dose used in this experiment (Avandia, 5 mg/kg/day) was used previously by Kang et al. (2013). STZ was obtained from Sigma-Aldrich Chemical Co. (St. Louis, MO, United States).

### The General Condition of the Rats and Biochemical Parameters

Each day, we monitored each rat with regards to energy level, diet, water intake, fur color, and general activity. Body weight (BW) and blood glucose (BG) were tested before modeling and every 2 weeks thereafter. The left kidneys of rats in the experimental groups were removed and weighed after cardiac puncture. After 6 weeks of drug intervention, the rats were anesthetized, and blood samples (5 ml) were drawn from the heart. A range of biochemical parameters were tested, including serum albumin (Alb), serum creatinine (Scr), and blood urea nitrogen (BUN). In addition, prior to sacrifice, we collected urinary samples from the four groups and used these samples to detect the ratio of urinary microalbumin to creatinine (UACR) and urinary *N*-acetyl-beta-*d*-glucosaminidase (UNAG); chromatometry was used to determine these parameters, as described previously (Liu et al., 2019).

### Histopathological Analysis, TUNEL Assays, and Immunohistochemistry

Kidney samples were fixed in 4% paraformaldehyde and embedded in paraffin. Then, sections (3 μm thick) were cut perpendicularly to the long axis of the kidney for histopathological analysis, immunohistochemistry (IHC), and terminal deoxynucleotidyl transferase dUTP nick end labeling (TUNEL) assay. For histopathological analysis, we used Periodic acid-Schiff (PAS) staining and Masson’s trichrome staining; stained sections were then investigated by light microscopy (LM). For the TUNEL assay, sections were treated with an *In Situ* Cell Death Detection Kit (Roche, Basel, Switzerland) in accordance with the manufacturer’s instructions. For IHC analysis, the paraffin-embedded kidney sections were deparaffinized in xylene and hydrated in a gradient series of alcohol concentrations, followed by water; the sections were subsequently placed in 3% H_2_O_2_ to eliminate endogenous peroxidase activity. Next, the sections were blocked with normal goat serum and incubated overnight at 4°C with anti-IL-6, anti-TGF-β, anti-collagen I, anti-vimentin, and anti-GRP78 antibodies (Abcam, Cambridge, United Kingdom). Finally, the sections were stained with a polymer horseradish peroxidase (HRP) detection system (ZSGB-BIO, Beijing, China) and counterstained with hematoxylin. The proportion (%) of positively stained renal tubulointerstitial area across 20 fields of view was then analyzed by Image-Pro Plus (IPP) 5.0 software (Media Cybernetic). Results of pathological analysis were confirmed by a pathologist.

### Western Blotting (WB) Analysis: *In Vivo*

WB analysis was performed as described previously (Tu et al., 2020). Renal tissues were isolated using phosphate-buffered saline containing protease inhibitors (PI), and sequentially solubilized with 1% Triton X-100, right inferior phrenic arteries (RIPA) buffer (0.1% sodium dodecyl sulfate (SDS), 1% sodium deoxycholate, 1% Triton X-100, 0.15 mol/L NaCl, and 0.01 mol/L ethylenediaminetetraacetic acid in 0.025 mol/L Tris-HCl, pH 7.2) with PI, and separated into Triton X-100-soluble (T), RIPA-soluble (R), and RIPA-insoluble (S) fractions. The RIPAI-insoluble fraction was solubilized with sodium dodecyl sulfate-polyacrylamide gel electrophoresis (SDS-PAGE) sample buffer (2% SDS, 10% glycerol, and 5% 2-mercaptoethanol in 0.0625 mol/L Tris-HCl, pH 6.8) (S fractions). Equal amounts of these sequentially solubilized fractions were subjected to SDS-PAGE with 7.5% or 10% acrylamide gels and then transferred onto a polyvinylidene fluoride membrane (Bio-Rad, Hercules, CA, United States) by electrophoretic trans-blotting for 30 min using a Trans-Blot SD system (Bio-Rad). After blocking with bovine serum albumin (BSA), the strips of membrane were exposed to anti-IL-6, TFG-β, VEGF, TLR4, α-SMA, vimentin, collagen I, Bax, Bcl-2, caspase12, GRP78, p-PERK, PERK, p-eIF2α, eIF2α, ATF4, CHOP, and glyceraldehyde-3-phosphate dehydrogenase (GAPDH) antibodies (Abcam, Cambridge, United Kingdom). Subsequently, these strips were washed and incubated with peroxidase-conjugated secondary antibodies for 1 h at room temperature. Finally, the bands were visualized by an alkaline phosphatase chromogen kit (5-bromo-4-chloro-3-indolil phosphate p-toluidine salt/nitro blue tetrazolium; Biomedica, AG, Staad, Switzerland). The density of the positive bands was quantified by densitometry (ATTO, Tokyo, Japan). Next, we determined the ratio of the densitometric signal of the target molecule to that of GAPDH.

### Cell Culture and Treatment

NRK-52E cells, a murine PTECs line, were kindly provided by Dr. Jian Yao (University of Yamanashi, Chuo, Japan) and cultured as described previously (Liu et al., 2018; Tu et al., 2020). In brief, the NRK-52E cells were cultured in Dulbecco’s modified Eagle’s medium/Ham’s F-12 (HyClone) supplemented with 5% fetal bovine serum (FBS; Gibco, Grand Island, NY, United States). The NRK-52E cells were then exposed to HG (30 mmol/L d-glucose) and 4-phenylbutyric acid (4-PBA, 5 mmol/L, ER stress inhibitor) with or without SKI at a concentration of 20 μg/ml or ROS at a concentration of 10 mmol/L for 24 h. For this experiment, the concentrations of SKI, ROS, and 4-PBA were described by Xu et al. (2016), Kang et al. (2013), and Min et al. (2018), respectively.

### Cell Viability Assessment

The viability of NRK-52E cells was assessed by the CCK-8 Kit (Beyotime, Shanghai, China). The cells were seeded into 96-well plates, with three replicate wells for each group, at a density of 1×10^4^ cells per well, with 100 μl of medium. After the cells were incubated for the indicated time period, 10 μl of CCK-8 solution was added to each well, followed by incubation for 2 h. The optical density (OD) was then determined at an absorbance of 450 nm, and the cell viability was calculated.

### WB Analysis: *In Vitro*

NRK-52E cells were administered with appropriate treatments for 24 h, respectively. Following treatment, cell lysates were separated by gel electrophoresis and blotted with antibodies against Bax, Bcl-2, caspase12, GRP78, p-PERK, PERK, p-eIF2α, eIF2α, ATF-4, CHOP, and GAPDH. The secondary antibody was HRP-conjugated anti-rabbit IgG antibody. WB analysis was then carried out on samples of cells from each group; for this, we used a protocol described in our previous publications (Tu et al., 2020; Liu et al., 2021).

### Statistical Analysis

The WB assessment was repeated at least thrice independently, and the individual data were subjected to a densitometric analysis. The data are expressed as the means ± standard deviation. The statistical analysis was performed by one way analysis of variance (ANOVA) on normally distributed data, with least significant difference (LSD) post-hoc test, or non-parametric Kruskal-Wallis if not. A *p*-value < 0.05 indicated a statistically significant difference.

## Results

### General Conditions, Renal Morphology, and Biochemical Parameters in the Rat Model of DKD Were all Improved by SKI and ROS

**FIGURE 1:**
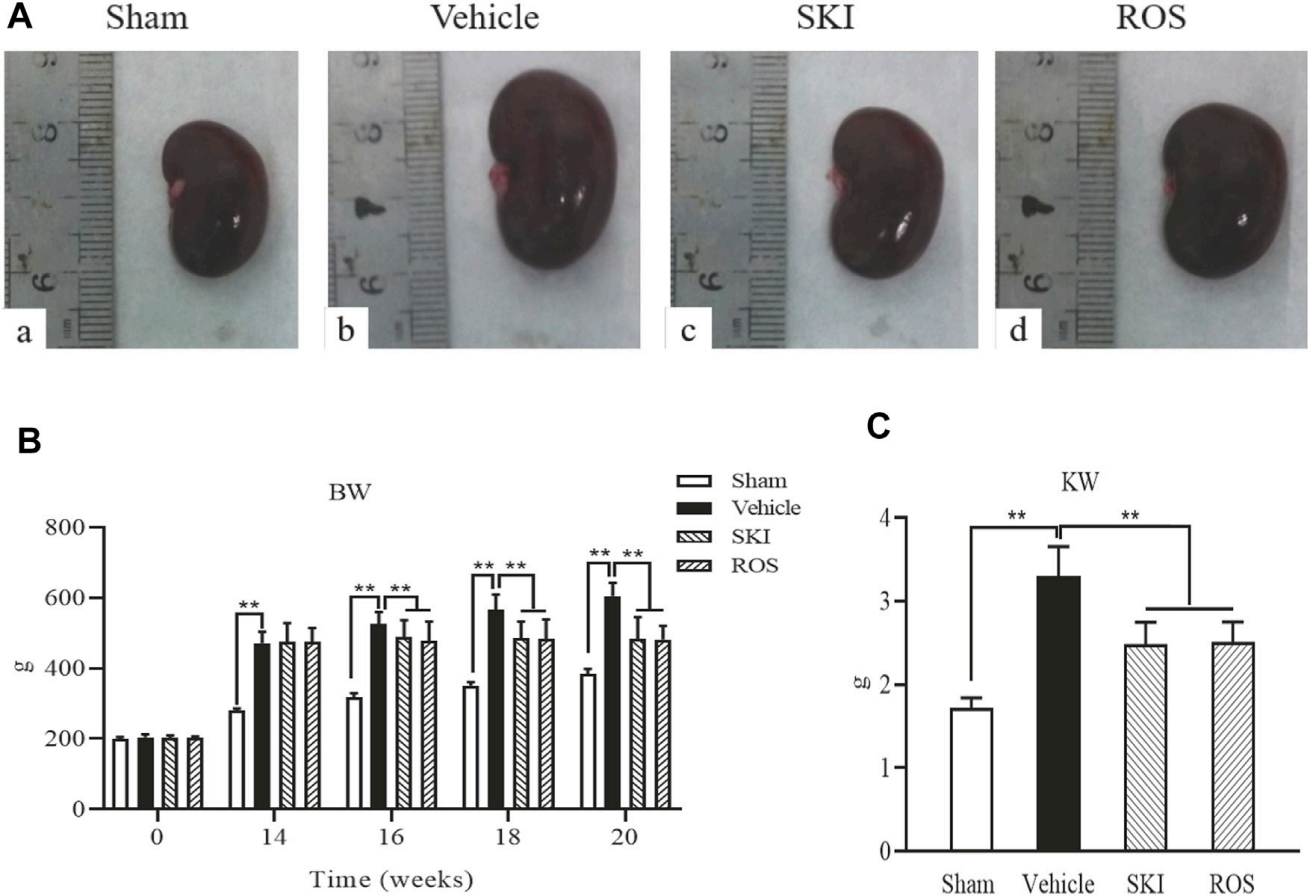
The effects of SKI and ROS on renal morphological appearance **(A)**, BW **(B)**, and KW **(C)** *in vivo*. Data are expressed as mean ± S.E. *p < 0.05, **p < 0.01. Abbreviations: BW, body weight; KW, kidney weight; ROS, rosiglitazone; SKI, Shenkang injection.

First, we compared the effects of SKI and ROS on the general condition, renal morphology, BW, and kidney weight (KW), in the rat model of DKD. Except for the Sham group of rats, the animals in the other three groups exhibited polyuria, polydipsia, polyphagia, low activity, dull fur, and BW gain; these effects occurred to differing degrees. The rats in the Vehicle group showed the most obvious effects. As shown in Figure 1, after sacrifice, we found that the kidneys of rats in the Sham group were moderated, while the kidneys of rats in the Vehicle group were swollen. The kidneys of rats treated with SKI or ROS were significantly ameliorated (for example, exhibited reduced less swelling and ischemia) when compared with rats from the Vehicle group (Figure 1A). In addition, the BW and KW of rats in the Vehicle group was obviously higher than those in the Sham group. The BW and KW of rats in the SKI and ROS groups was lower than those of rats in the Vehicle group (Figures 1B,C).

**FIGURE 2:**
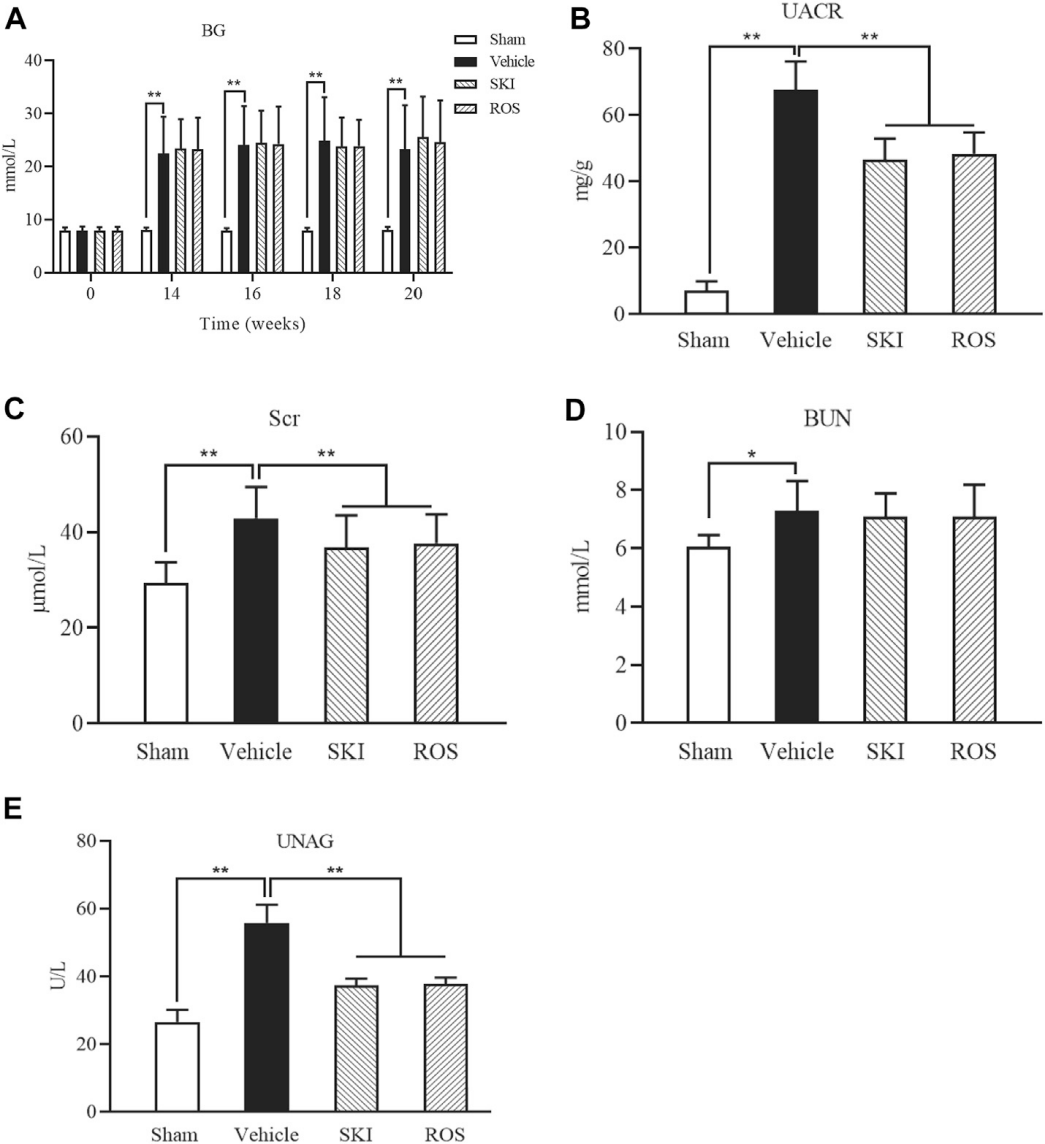
The effects of SKI and ROS on BG **(A)**, UACR **(B)**, Scr **(C)**, BUN **(D)**, and UNAG **(E)** *in vivo*. Data are expressed as mean ± S.E. **p < 0.01. Abbreviations: BG, blood glucose; UACR, urinary microalbumin to creatinine; Scr, serum creatinine; BUN, blood urea nitrogen; UNAG, urinary *N*-acetyl-beta-*d*-glucosaminidase; ROS, rosiglitazone; SKI, Shenkang injection.

Next, we compared the effects of SKI and ROS on BG and UACR in the DKD model rats. As shown in Figure 2, following the induction of renal injury, the levels of BG in the Vehicle group rats increased and were maintained at high levels by consecutive subcutaneous injections of Novolin N. Furthermore, the levels of BG in the SKI and ROS groups of rats also remained at high levels following the induction of renal injury for 6 weeks. There were no significant differences in the levels of BG when compared between the SKI and ROS groups of rats (Figure 2A). Moreover, 6 weeks after the induction of renal injury, the levels of UACR of rats in the Vehicle group had reached an abnormal level of 67.79 ± 8.35 mg/g; these were significantly higher than those in the Sham group of rats. After the treatment with SKI or ROS for 6 weeks, the levels of UACR in the SKI and ROS group rats were significantly lower than those in rats from the Vehicle group (Figure 2B).

Next, we investigated the effects of SKI and ROS on BUN, Scr, and UNAG, in the rat model of DKD. As shown in Figure 2, 6 weeks after the induction of renal injury, the levels of BUN, Scr, and UNAG in the Vehicle group were significantly higher than those in the Sham group. Following 6 weeks of SKI and ROS treatment, the levels of Scr and UNAG in the rat model of DKD were significantly lower than those in the Vehicle group of rats. However, the levels of BUN in the SKI and ROS groups of rats did not change significantly after SKI or ROS treatment (Figures 2C–E).

These data indicated that SKI and ROS partially improved the general condition, renal morphology, and biochemical parameters of the rat model of DKD, including BW, KW, UACR, Scr, and UNAG.

### Renal Tubular Injury in the Rat Model of DKD Was Ameliorated by SKI and ROS

**FIGURE 3:**
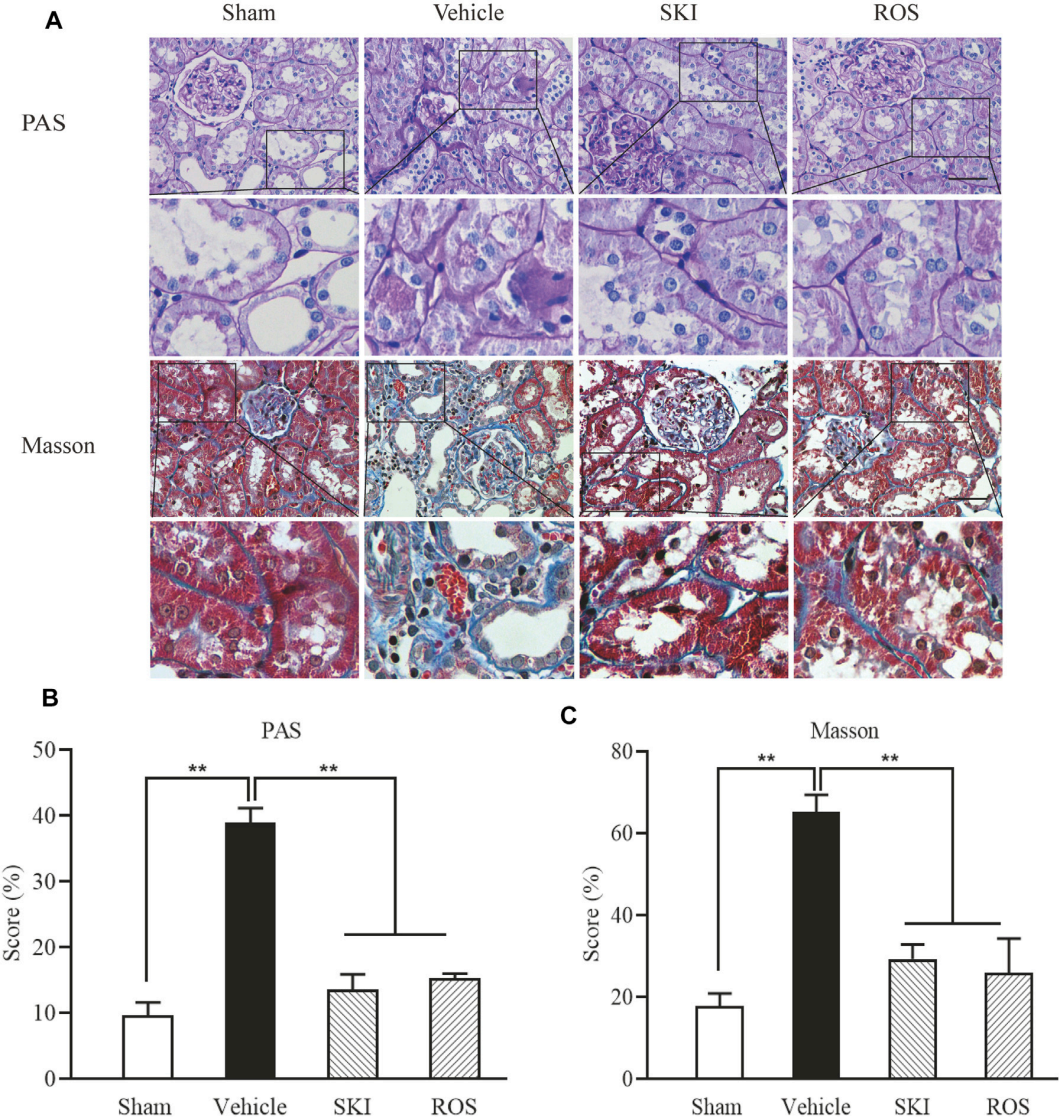
The effects of SKI and ROS on tubulointerstitial pathological changes *in vivo*. **(A)** PAS and Masson staining in the kidney (× 200); **(B)** The rate of ECM/interstitial area; **(C)** The rate of collagen/interstitial area. Scale bar = 100 μm. Data are expressed as mean ± S.E. **p < 0.01. Abbreviations: PAS, Periodic acid-Schiff; ECM, extracellular matrix; ROS, rosiglitazone; SKI, Shenkang injection.

Next, we investigated the effects of SKI and ROS on tubulointerstitial pathological changes in the rat model of DKD. As shown in Figure 3, 6 weeks after the induction of renal injury, LM showed that there were obvious pathological changes in the Vehicle group of rats when compared to those in the Sham group, including epithelial cell edema and hypertrophy, vacuolar lesions in the renal tubules, tubular obliteration, and irregular morphology; we also observed slight deposition of collagen in the renal interstitium. After 6 weeks of SKI or ROS treatment, the degree of tubulointerstitial pathological changes in the rat model of DKD were significantly improved when compared to those in the Vehicle group, including epithelial cell edema and hypertrophy, vacuolar lesions in the renal tubules, tubular obliteration and irregular morphology, and collagen deposition in the renal interstitium. Notably, we did not observe obvious extracellular matrix (ECM) accumulation in the renal interstitium in the DKD rats.

**FIGURE 4:**
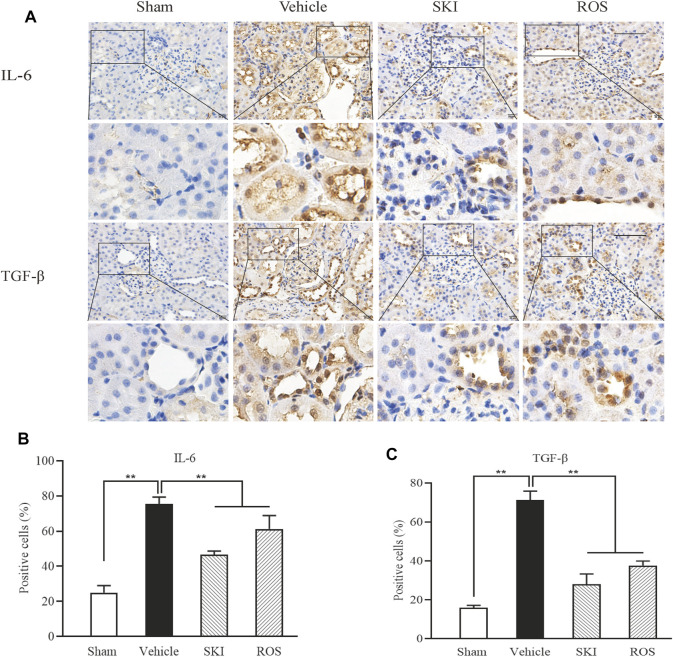
The effects of SKI and ROS on the expression levels of IL-6 and TGF-β *in vivo*. **(A)** Immunostaining of IL-6 and TGF-β in the kidney (× 200); **(B, C)** The number of positively stained cells. Scale bar = 100 μm. Data are expressed as mean ± S.E. **p < 0.01. Abbreviations: IL-6, interleukin-6; TGF-β, transforming growth factor-β; ROS, rosiglitazone; SKI, Shenkang injection.

**FIGURE 5:**
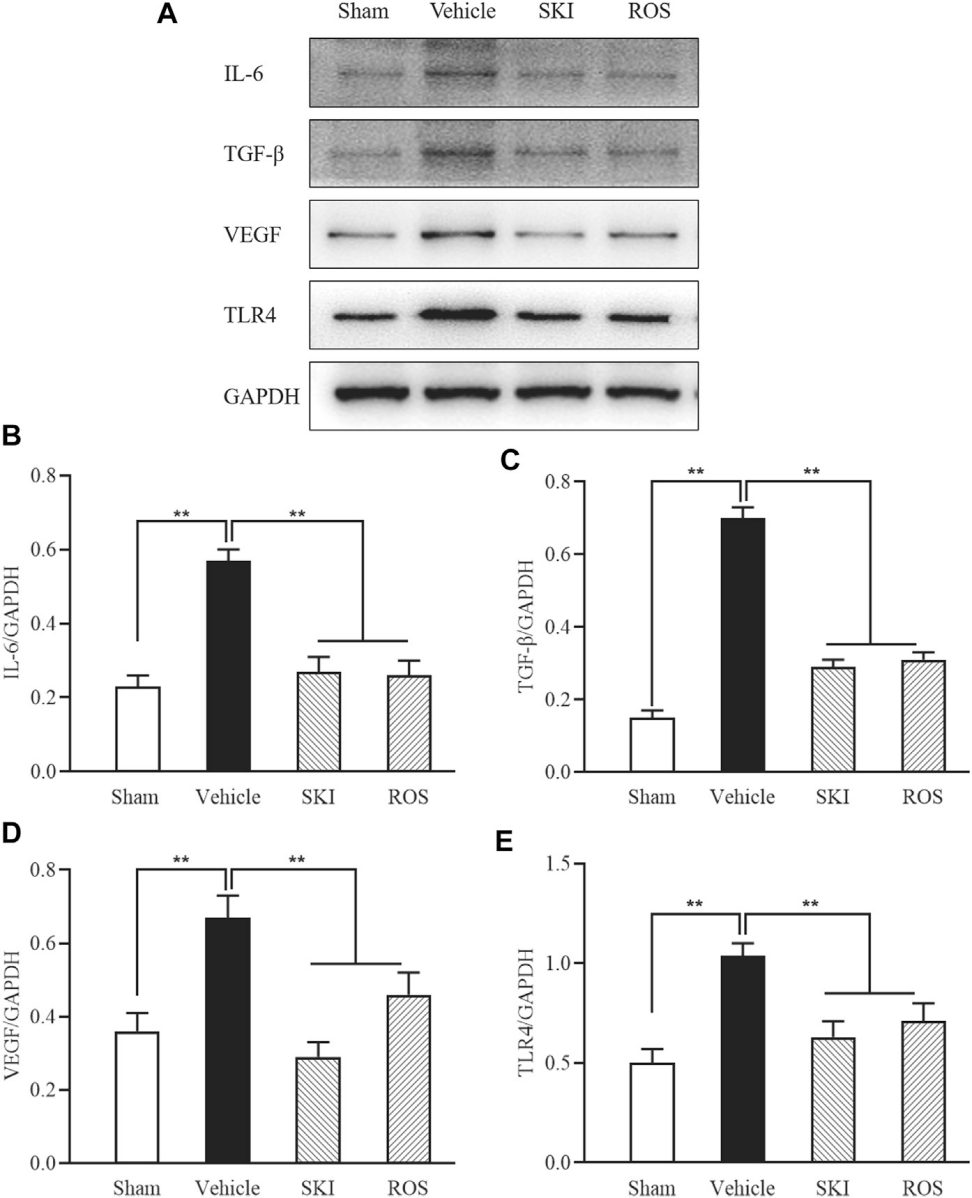
The effects of SKI and ROS on the protein expression levels of IL-6, TGF-β, VEGF, and TLR4, *in vivo*. **(A)** WB analysis of IL-6, TGF-β, VEGF, and TLR4, in the kidney; **(B–E)** The rate of IL-6, TGF-β, VEGF, and TLR4 to GAPDH, respectively. Data are expressed as mean ± S.E. **p < 0.01. Abbreviations: IL-6, interleukin-6; TGF-β, transforming growth factor-β; VEGF, vascular endothelial growth factor; TLR4, till-like receptor 4; GAPDH, glyceraldehyde-3-phosphate dehydrogenase; ROS, rosiglitazone; SKI, Shenkang injection.

Next, we investigated the effects of SKI and ROS on the expression levels of a range of injury markers in the renal tubules of DKD model rats by IHC staining and WB analysis, including IL-6, TGF-β, VEGF, and TLR4. As shown in Figures 4, 5, compared with the Sham group of rats, 6 weeks after the induction of renal injury, we observed significant immunostaining of IL-6 and TGF-β in the renal tubular epithelial cells and increased protein levels of IL-6, TGF-β, VEGF, and TLR4 in the kidneys of the Vehicle group rats. Compared to rats in the Vehicle group, those in the SKI and ROS groups showed significant reductions in these parameters after 6 weeks of the treatment.

Collectively, these data indicated that SKI and ROS significantly ameliorated various aspects of renal tubular injury in the rat model of DKD, including tubulointerstitial pathological changes and the expression levels of various markers of renal tubular injury.

### Renal Tubular EMT in the Rat Model of DKD Was Attenuated by SKI and ROS

**FIGURE 6:**
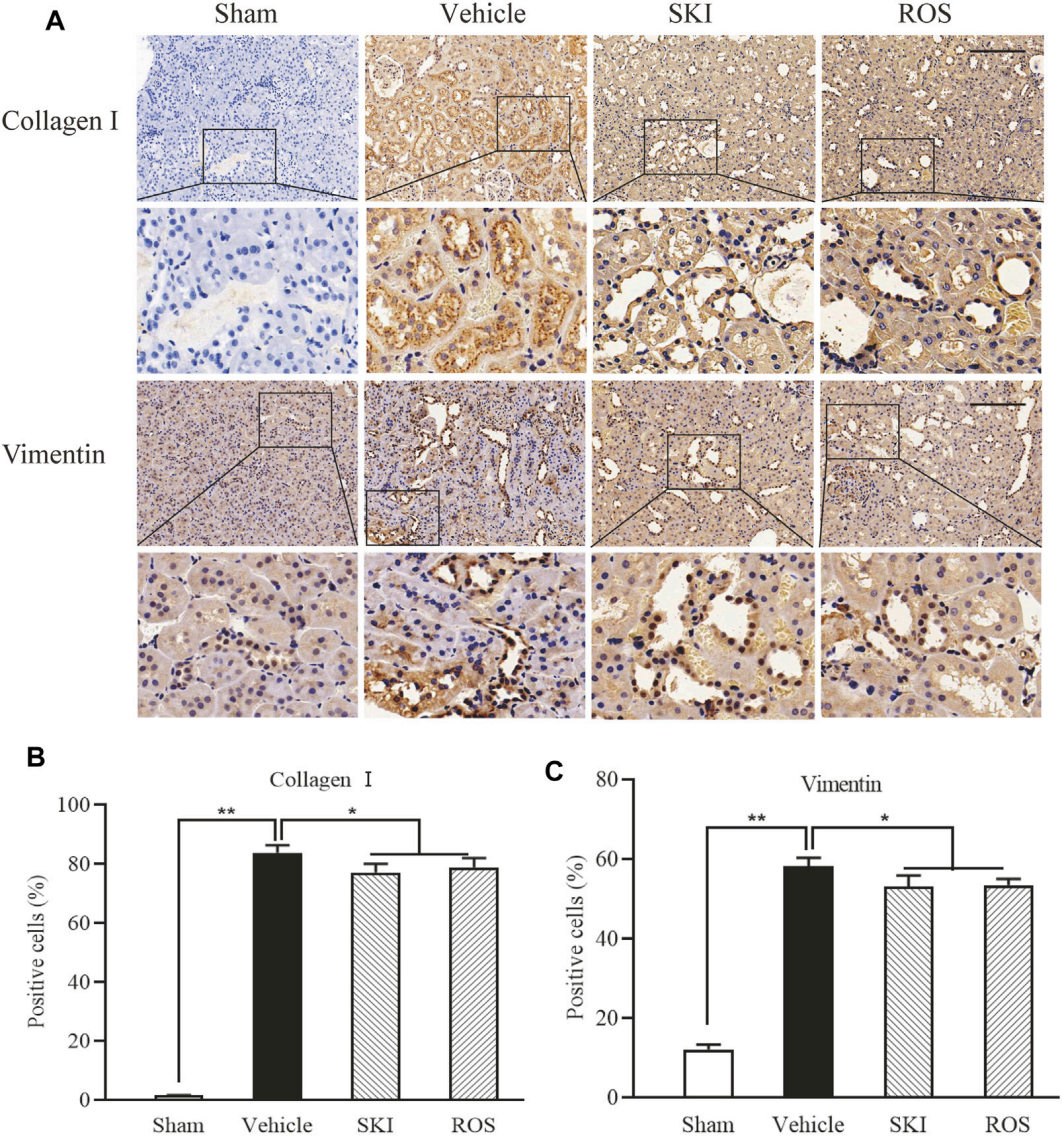
The effects of SKI and ROS on the expression levels of collagen I and vimentin *in vivo*. **(A)** Immunostaining of collagen I and vimentin in the kidney (× 200); **(B, C)** The number of positively stained cells. Scale bar = 100 μm. Data are expressed as mean ± S.E. *p < 0.05, **p < 0.01. Abbreviations: collagen I, collagen type I; ROS, rosiglitazone; SKI, Shenkang injection.

**FIGURE 7:**
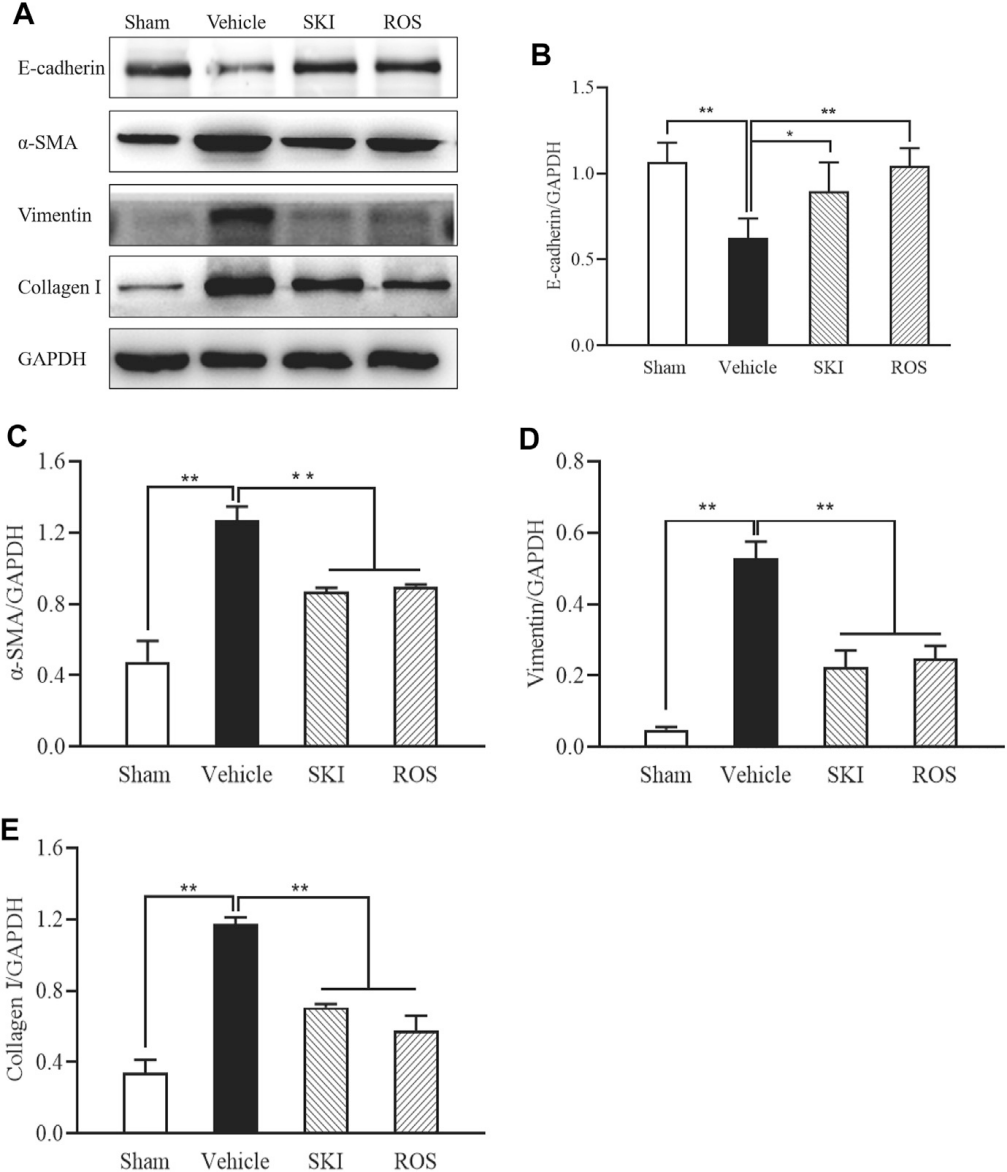
The effects of SKI and ROS on the protein expression levels of E-cadherin, α-SMA, vimentin, and collagen I, *in vivo*. **(A)** WB analysis of α-SMA, vimentin, and collagen I, in the kidney; **(B–E)** The rate of α-SMA, vimentin, and collagen I to GAPDH, respectively. Data are expressed as mean ± S.E. **p < 0.01. Abbreviations: α-SMA, α-smooth muscle actin; collagen I, collagen type I; WB, Western blot; GAPDH, glyceraldehyde-3-phosphate dehydrogenase ROS, rosiglitazone; SKI, Shenkang injection.

Next, we used IHC and WB analysis to investigate the effects of SKI and ROS on various markers of renal tubular EMT, including E-cadherin, collagen I, vimentin, and α-SMA in the kidneys of DKD rats. As shown in Figures 6, 7, 6 weeks after the induction of renal injury, compared with the Sham group of rats, there was obvious immunostaining of collagen I and vimentin in the renal tubular epithelial cells of rats in the Vehicle group. This was accompanied by increased protein expression levels of collagen I, vimentin, and α-SMA, as well as decreased protein expression level of E-cadherin in the kidneys. There were significant reversions in these parameters in the SKI and ROS groups of rats after 6 weeks of SKI or ROS treatment when compared to those of the Vehicle group of rats.

These data showed that SKI and ROS significantly attenuated renal tubular EMT in the rat model of DKD.

### Apoptosis in the Renal Tubular Cells Was Alleviated by SKI and ROS *in Vivo* and *in Vitro*.

**FIGURE 8:**
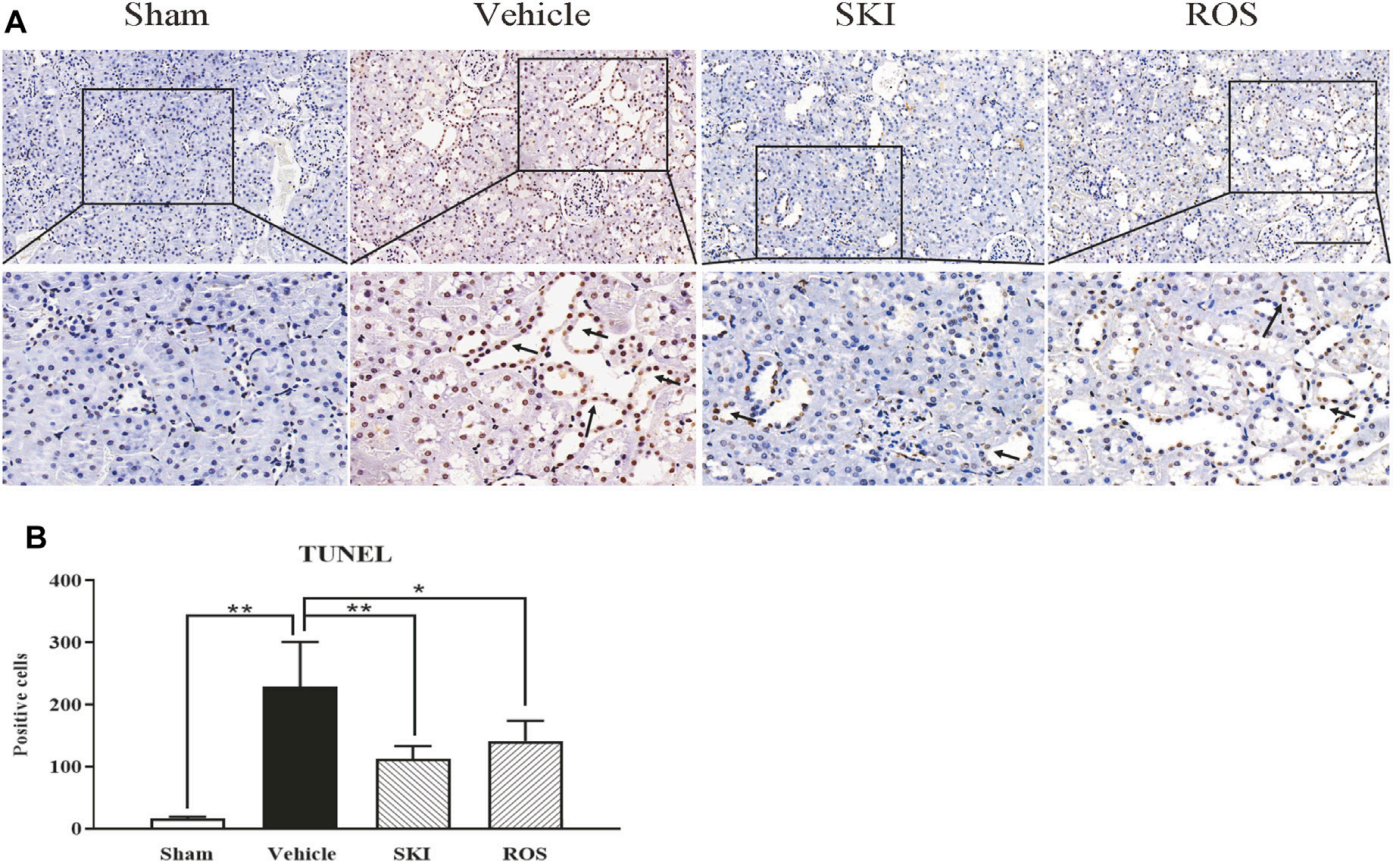
The effects of SKI and ROS on TUNEL staining *in vivo*. **(A)** TUNEL staining in the kidney (× 200); **(B)** The number of positively stained cells. Scale bar = 100 μm. Data are expressed as mean ± S.E. *p < 0.05, **p < 0.01. Abbreviations: TUNEL, terminal deoxynucleotidyl transferase dUTP nick end labeling; ROS, rosiglitazone; SKI, Shenkang injection.

**FIGURE 9:**
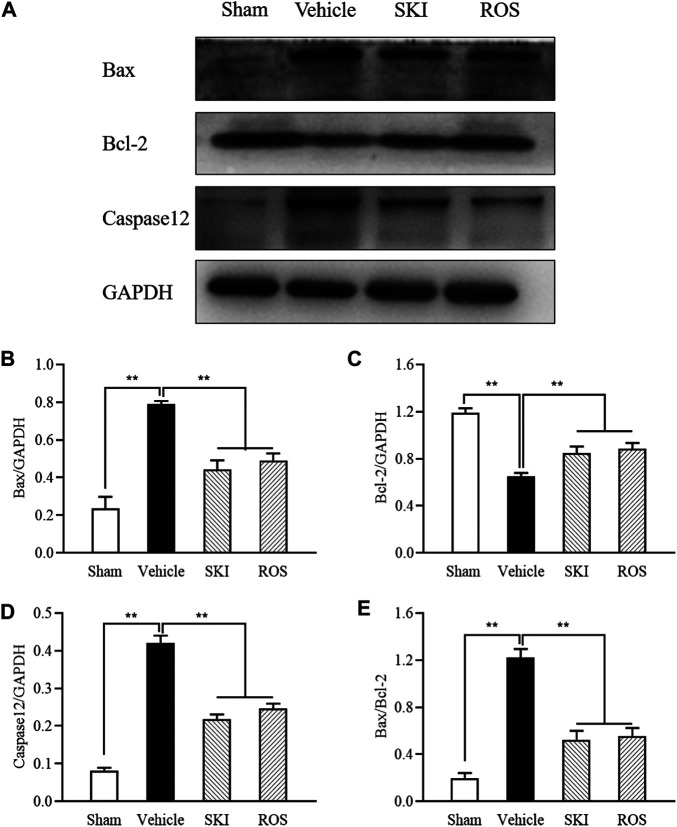
The effects of SKI and ROS on the protein expression levels of Bax, Bcl-2, and caspase12, *in vivo*. **(A)** WB analysis of Bax, Bcl-2, and caspase12, in the kidney; **(B–E)** The rate of Bax, Bcl-2 and caspase12 to GAPDH, respectively, and the rate of Bax to Bcl-2. Data are expressed as mean ± S.E. **p < 0.01. Abbreviations: WB, Western blot; GAPDH, glyceraldehyde-3-phosphate dehydrogenase; ROS, rosiglitazone; SKI, Shenkang injection.

Next, we used TUNEL staining and WB analysis to investigate the effects of SKI and ROS on apoptosis in the renal tubular cells as indicated by staining in the renal tubular epithelial cells and changes in the protein expression levels of Bax, Bcl-2, and caspase12 in the kidneys of the rat model of DKD. As shown in Figures 8, 9, 6 weeks after the induction of renal injury, compared with the Sham group of rats, there was obvious TUNEL staining of the renal tubular epithelial cells in the Vehicle group of rats, along with increased protein expression levels of Bax and caspase12; the level of Bcl-2 was significantly reduced in the kidneys of the Vehicle group of rats. These parameters all showed significant improvement in the SKI and ROS groups of rats after 6 weeks of the treatment when compared to those of the Vehicle group of rats.

**FIGURE 10:**
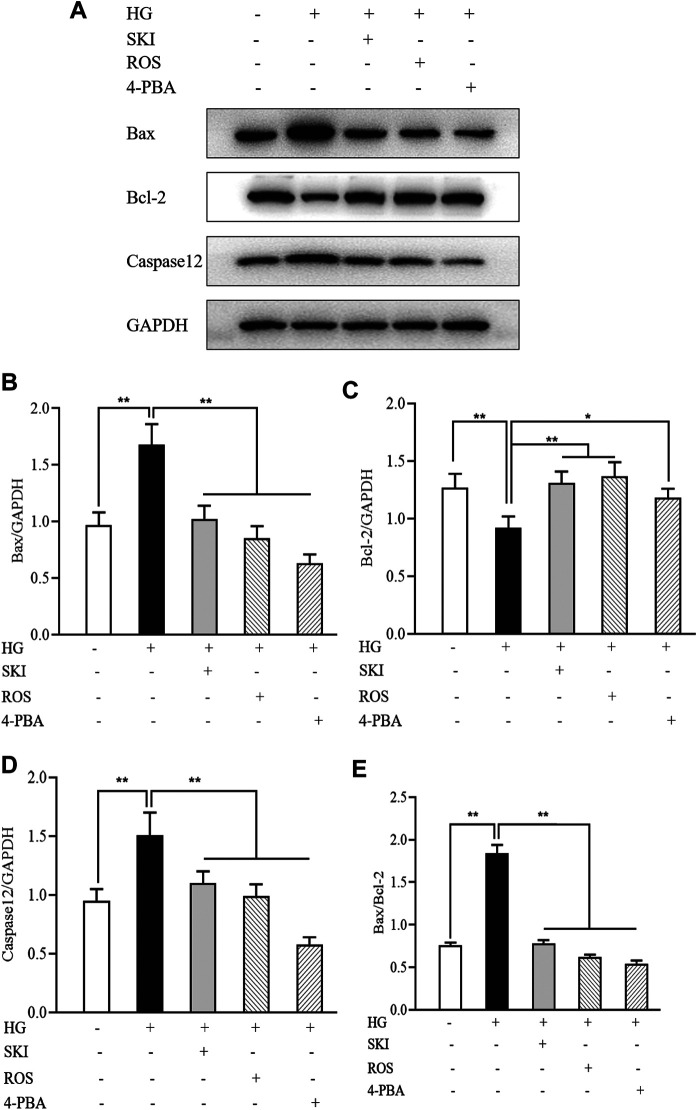
The effects of SKI and ROS on the protein expression levels of Bax, Bcl-2, and caspase12, *in vitro*. **(A)** WB analysis of Bax, Bcl-2, and caspase12, in NRK-52E cells exposed to HG and 4-PBA with or without SKI or ROS for 24 h; **(B–E)** The rate of Bax, Bcl-2, and caspase12, to GAPDH, respectively, and the rate of Bax to Bcl-2. Data are expressed as mean ± S.E. *p < 0.05, **p < 0.01. Abbreviations: WB, Western blot; HG, high glucose; 4-PBA, 4-phenylbutyric acid; GAPDH, glyceraldehyde-3-phosphate dehydrogenase; ROS, rosiglitazone; SKI, Shenkang injection.

To investigate whether SKI and ROS can reduce apoptosis in the renal tubular cells *in vitro*, we determined the protein expression levels of Bax, Bcl-2, and caspase12 in NRK-52E cells exposed to HG with or without SKI or ROS for 24 h. Prior to the formal cellular experiments, we determined the cytotoxicity of SKI or ROS on NRK-52E cells with a CCK-8 kit. As shown in Supplemental Figure S3, cellular viability was significantly reduced when treated with higher concentrations of SKI (40 μg/ml) and ROS (200 nmol/L) when compared to a 20 μg/ml concentration of SKI and a 100 nmol/L concentration of ROS. Based on these results, we selected safe and effective concentrations for both SKI (20 μg/ml) and ROS (100 nmol/L). Figure 10 shows that the protein expression levels of Bax and caspase 12 were significantly higher, while the level of Bcl-2 was significantly lower in NRK-52E cells exposed to HG when compared to control cells. The treatment with SKI or ROS significantly ameliorated these changes in NRK-52E cells exposed to HG when compared to HG treatment alone. In addition, we also observed improvements in the protein expression levels of Bax, Bcl-2, and caspase12 in NRK-52E cells exposed to HG and 4-PBA (an inhibitor of ER stress) when compared to cells treated with HG alone.

Collectively, these data showed that SKI and ROS alleviated apoptosis in the renal tubular cells both *in vivo* and *in vitro*.

### ER Stress in Renal Tubular Cells Was Inhibited by SKI and ROS *in Vivo* and *in Vitro*

**FIGURE 11:**
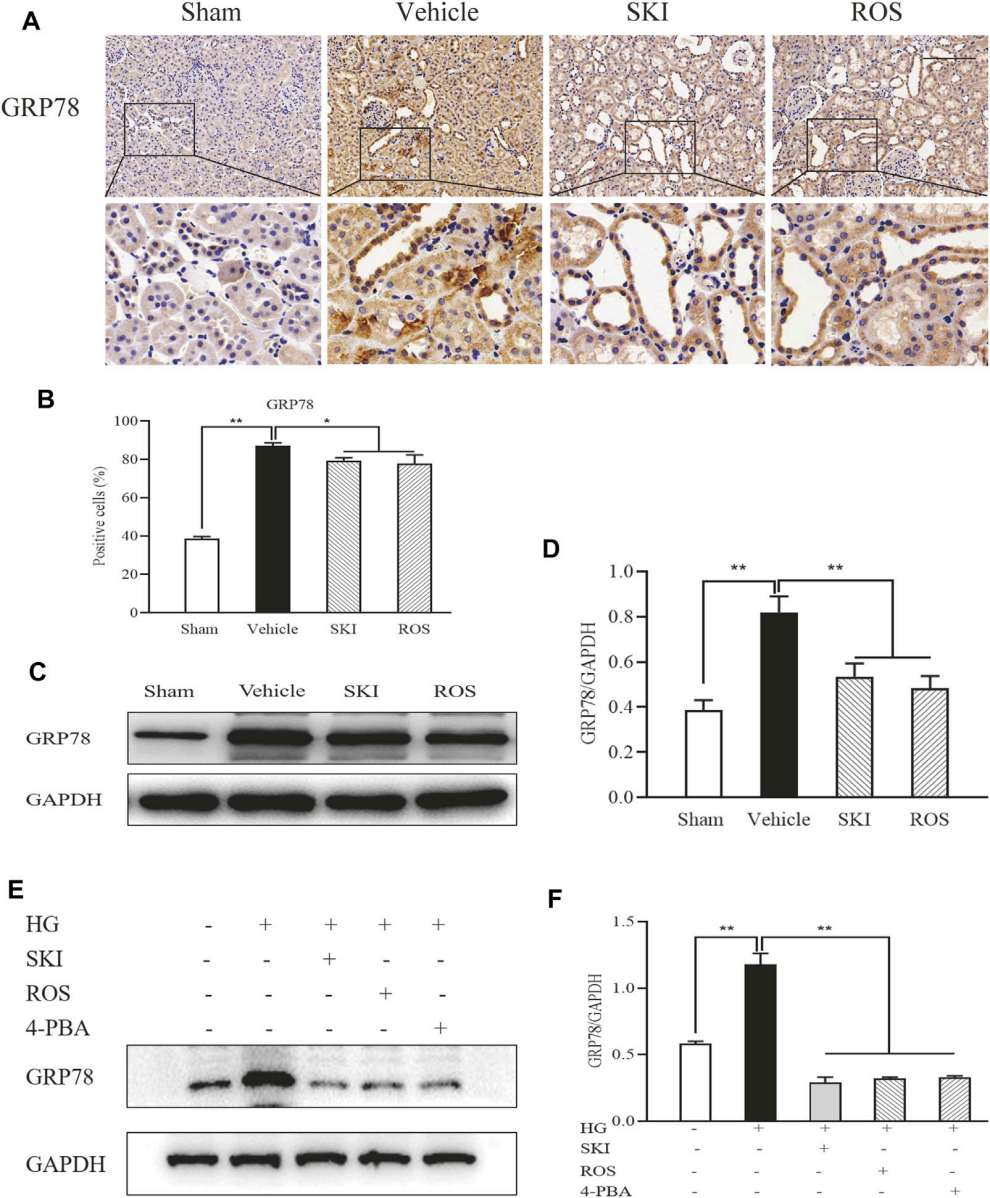
The effects of SKI and ROS on the expression levels of GRP78 *in vivo* and *in vitro*. **(A)** Immunostaining of GRP78 in the kidney (× 200); **(B)** The number of positively stained cells; **(C)** WB analysis of GRP78 in the kidney; **(D)** The rate of GRP78 to GAPDH; **(E)** WB analysis of GRP78 in NRK-52E cells exposed to HG and 4-PBA with or without SKI or ROS for 24 h. **(F)** The rate of GRP78 to GAPDH. Scale bar = 100 μm. Data are expressed as mean ± S.E. *p < 0.05, **p < 0.01. Abbreviations: GRP78, glucose-regulated protein 78; WB, Western blot; GAPDH, glyceraldehyde-3-phosphate dehydrogenase; HG, high glucose; 4-PBA, 4-phenylbutyric acid; ROS, rosiglitazone; SKI, Shenkang injection.

ER stress is an important factor that acts upstream of apoptosis (Kitamura, 2008b; Kitamura, 2009). Therefore, we evaluated the expression of GRP78 as a marker of ER stress in the kidneys of DKD rats by IHC staining and WB analysis. As shown in Figure 11, 6 weeks after the induction of renal injury, compared with the Sham group of rats, there was obvious GRP78 immunostaining in the renal tubular epithelial cells and increased protein expression levels of GRP78 in the kidneys of the Vehicle group. SKI and ROS treatment led to a significant reduction in these effects when compared to those in the Vehicle group.

Next, we tested the protein expression level of GRP78 in NRK-52E cells exposed to HG with or without SKI or ROS for 24 h. Figure 11 shows that when compared with control cells, the NRK-52E cells exposed to HG exhibited significantly higher expression levels of GRP78 protein; when treated with SKI or ROS, NRK-52E cells exposed to HG exhibited a significant reduction in the levels of GRP78 protein when compared to HG treatment alone. In addition, we observed that the protein expression level of GRP78 was lower in NRK-52E cells exposed to HG and 4-PBA than those treated with HG alone.

These data showed that SKI and ROS inhibited renal tubular ER stress *in vivo* and *in vitro*.

### Activation of the PERK-eIF2α-ATF4-CHOP Signaling Pathway Was Suppressed by SKI and ROS *in Vivo* and *in Vitro*.

**FIGURE 12:**
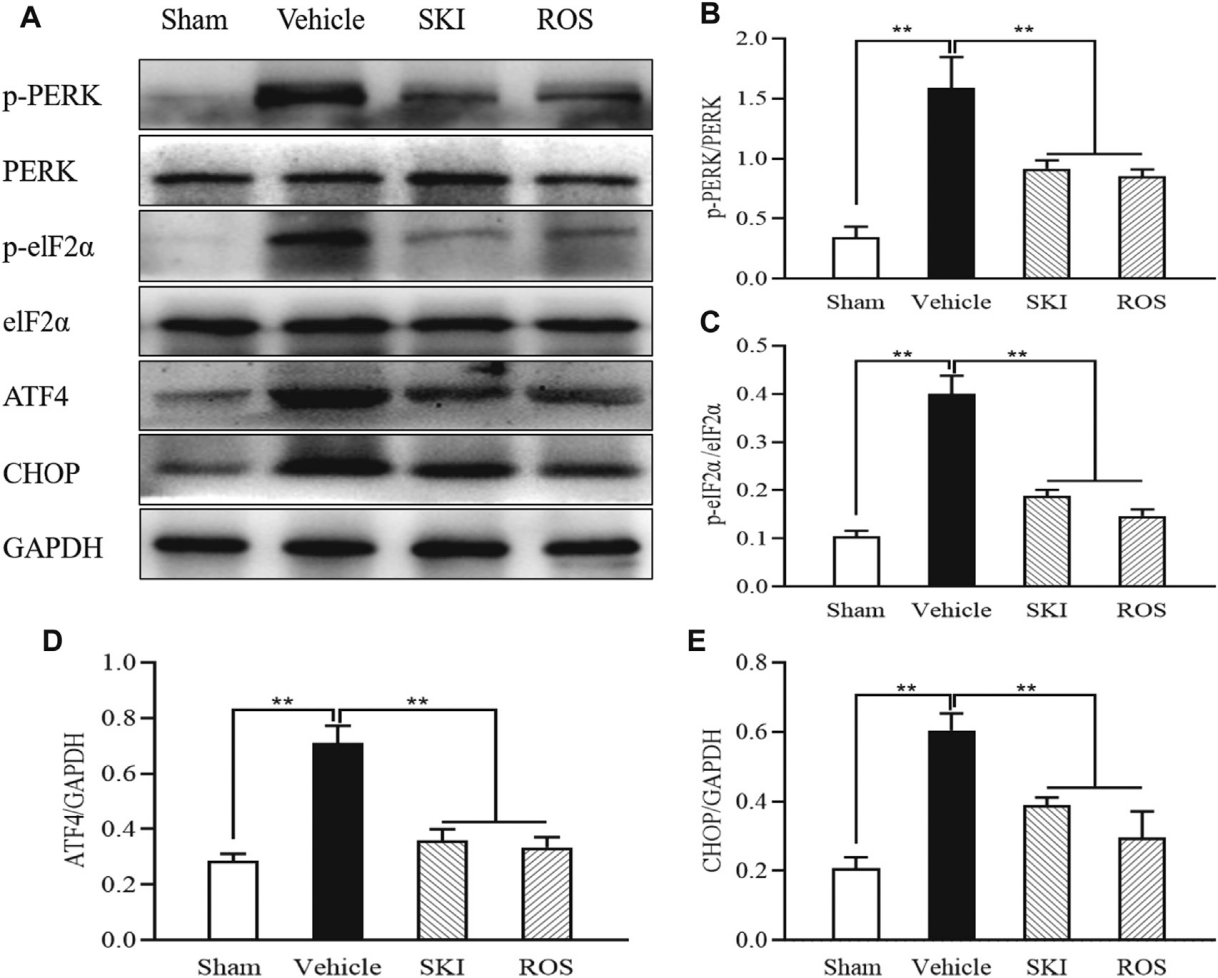
The effects of SKI and ROS on the expression levels of key signaling molecules in the PERK-eIF2α-ATF4-CHOP pathway *in vivo*. **(A)** WB analysis of p-PERK, PERK, p-eIF2α, eIF2α, ATF4, and CHOP, in the kidney; **(B–E)** The rate of p-PERK to PERK and p-eIF2α to eIF2α, and the rate of ATF4 and CHOP to GAPDH, respectively. Data are expressed as mean ± S.E. **p < 0.01. Abbreviations: PERK, double-stranded RNA (PKR)-activated protein kinase-like eukaryotic translation initiation factor 2α (eIF2α) kinase; eIF2α, double-stranded RNA (PKR)-activated protein kinase-like eukaryotic translation initiation factor 2α; ATF4, activating transcription factor 4; CHOP, C/EBP homologous protein; WB, Western blot; GAPDH, glyceraldehyde-3-phosphate dehydrogenase; ROS, rosiglitazone; SKI, Shenkang injection.

PERK-eIF2α-ATF4-CHOP signaling is an important regulatory mechanism in ER stress (Kitamura, 2008a; Kang et al., 2017). Thus, we used WB analysis to investigate the expression levels of key signaling molecules in the renal PERK-eIF2α-ATF4-CHOP pathway. Figure 12 shows that the protein expression levels of p-PERK, p-eIF2α, ATF4, and CHOP, in the kidneys of the Vehicle group of rats were significantly higher than those in the Sham group of rats. In comparison with the Vehicle group of rats, those in the SKI or ROS groups exhibited significant reductions in the protein expression levels of p-PERK, p-eIF2α, ATF4, and CHOP, in the kidneys.

**FIGURE 13:**
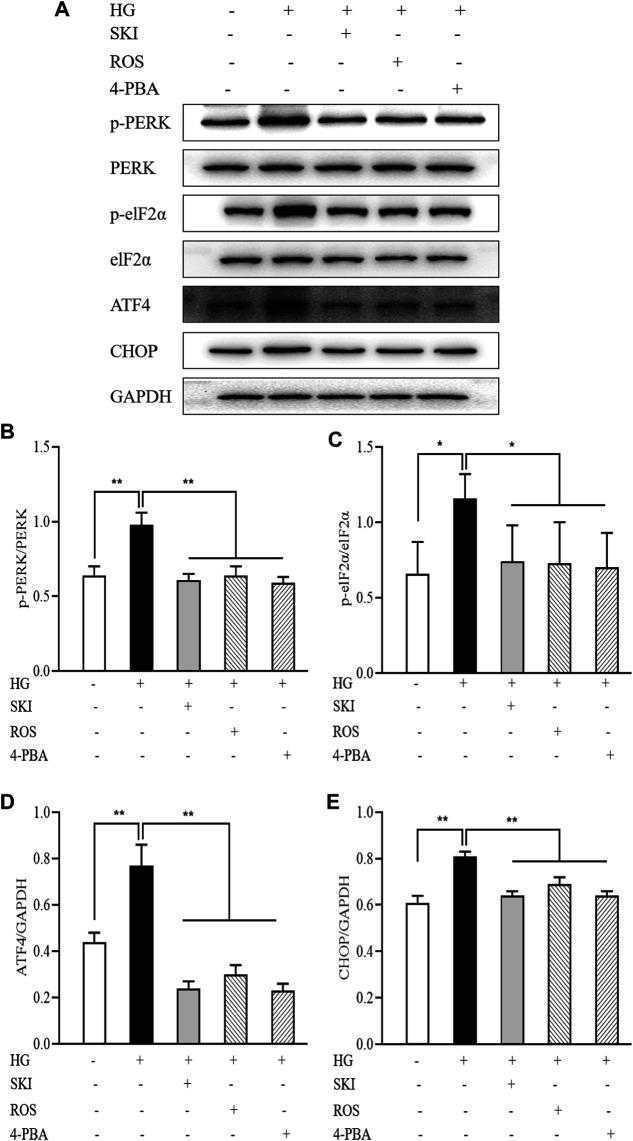
The effects of SKI and ROS on the expression levels of key signaling molecules in the PERK-eIF2α-ATF4-CHOP pathway *in vitro*. **(A)** WB analysis of p-PERK, PERK, p-eIF2α, eIF2α, ATF4, and CHOP, in NRK-52E cells exposed to HG and 4-PBA with or without SKI or ROS for 24 h **(B–E)** The rate of p-PERK to PERK and p-eIF2α to eIF2α, and the rate of ATF4 and CHOP to GAPDH, respectively. Data are expressed as mean ± S.E. *p < 0.05, **p < 0.01. Abbreviations: PERK, double-stranded RNA (PKR)-activated protein kinase-like eukaryotic translation initiation factor 2α (eIF2α) kinase; eIF2α, double-stranded RNA (PKR)-activated protein kinase-like eukaryotic translation initiation factor 2α; ATF4, activating transcription factor 4; CHOP, C/EBP homologous protein; WB, Western blot; GAPDH, glyceraldehyde-3-phosphate dehydrogenase; HG, high glucose; 4-PBA, 4-phenylbutyric acid; ROS, rosiglitazone; SKI, Shenkang injection.

Figure 13 shows that the protein expression levels of p-PERK, p-eIF2α, ATF4, and CHOP, in NRK-52E cells exposed to HG were significantly higher than those in the control cells. In comparison with HG treatment alone, the protein expression levels of p-PERK, p-eIF2α, ATF4, and CHOP, in NRK-52E cells exposed to SKI or ROS were all significantly lower. In addition, we also observed significantly lower protein expression levels of p-PERK, p-eIF2α, ATF4, and CHOP, in NRK-52E cells exposed to HG and 4-PBA than those treated with HG alone.

These data showed that SKI and ROS suppressed activation of the PERK-eIF2α-ATF4-CHOP signaling pathway, both *in vivo* and *in vitro*.

## Discussion

In 1999, Gilbert and Cooper proposed the non-glomerular mechanisms that may be involved in the tubular injury caused by DM and emphasized that the renal tubulointerstitial damage was more extensive in such cases than could be explained by glomerular disease alone (Gilbert and Cooper, 1999). An increasing body of evidence now suggests a primary role for PTECs in the creation of lesions and dysfunction in the kidney nephrons, and has led to this condition being referred to by the independent term diabetic tubulopathy or “DT” (Tang et al., 2011; Vallon, 2011). At present, it is generally accepted that hyperglycemia is an important causative factor in the pathogenesis of DKD and that this condition exerts both proinflammatory and profibrotic effects on PTECs. *In vitro* and *in vivo* studies have shown that the exposure of PTECs to HG conditions promotes proinflammatory, profibrotic, and angiogenic responses that are associated with tubulointerstitial pathological changes and the overexpression of several deleterious factors in the renal tubules, including IL-6, TGF-β, VEGF, and TLR4 (Alicic et al., 2017; Chen et al., 2017). Therefore, in this study, we first tried to establish a rat model of DKD that involved renal tubular injury. Our results showed that we successfully established a rat model of DKD and that these animals exhibited hyperglycemia; expressed abnormal levels of UACR and UNAG; expressed increased levels of Scr and BUN; and exhibited obvious tubulointerstitial pathological changes, including epithelial cell edema and hypertrophy, renal tubular vacuolar lesions, tubular obliteration and irregular morphology, and slight deposition of collagen in the renal interstitium. Furthermore, more importantly, we detected significantly higher expression levels of IL-6, TGF-β, VEGF, and TLR4 in the kidneys of these DKD rat models; these factors are all known to cause deleterious effects in terms of kidney function. We thereby considered that our rat model of DKD should be helpful to understand the mechanisms underlying tubular injury and to identify novel therapeutic drugs for DT.

In our previous studies, we reported that SKI, a modern preparation of Chinese patent medicine, was able to reduce TIF in obstructive nephropathy (Liu et al., 2019). In the present study, we established a rat model of DKD with tubular injury by performing unilateral nephrectomy and injecting STZ. Following the induction of renal injury, we treated the DKD model rats for 6 weeks with SKI and ROS. We found that these treatments led to an improvement in the general condition of the rats, BW, KW, biochemical parameters (including UACR, Scr, and UNAG), tubulointerstitial pathological changes, and the expression levels of various markers of renal tubular injury (including IL-6, TGF-β, VEGF, and TLR4). The beneficial effects of SKI were similar to those of ROS, a potent insulin sensitizer in the clinic.

EMT is an essential process during the early stages of DKD, not only in the pathogenesis of TIF, but also in the mechanisms that lead to glomerulosclerosis (Loeffler and Wolf, 2015). It is well known that one of the recognized hallmarks of EMT is a clear gain in certain properties of the mesenchymal cells, including the expression of vimentin, α-SMA, and collagen I (Liu, 2004). Our previous study demonstrated that SKI can attenuate fibrotic tubulointerstitial damage in a rat model of unilateral ureteral obstruction (UUO) by blocking pericyte-myofibroblast transition, a process that is known to be similar to EMT (Liu et al., 2019). In this study, we examined a range of EMT markers (including vimentin, α-SMA, and collagen I) in diabetic kidneys to investigate whether SKI and ROS can alleviate renal tubular EMT. Our results revealed that the immunostaining of collagen I and vimentin in the renal tubular epithelial cells, and increased protein expression levels of collagen I, vimentin, and α-SMA, in the kidneys of the DKD rats were significantly reduced following 6 weeks of treatment with SKI or ROS. We therefore considered that SKI could significantly attenuate renal tubular EMT in the rat model of DKD in a manner that was similar to ROS.

An accumulating body of evidence, from both clinical scenarios and experimental animal models, supports the fact that hyperglycemia induces apoptosis through ER stress in tubular cells, at least in part, and that this process contributes to the pathogenesis of DT (Kang et al., 2020). ER stress generally occurs under normal physiological and pathological conditions and represents an important inducer of cell apoptosis (Cybulsky, 2017). In the present study, we used TUNEL-positive tubular cells and abnormal expression levels of Bax, Bcl-2, and GRP78, as well established markers of apoptosis and ER stress. Furthermore, caspase12 is exclusively localized in the ER and is ubiquitously and constitutively expressed. However, unlike other caspases, caspase12 is specifically activated by insults that induce ER stress and not by other death stimuli (Cunard, 2015). In the present study, our data indicated that 6 weeks of SKI and ROS treatment led to significant improvements in the number of TUNEL-positive tubular cells, and ameliorated the high protein expression levels of Bax and caspase12, and the reduced protein expression levels of Bcl-2, in the kidneys of DKD rats. In addition, to confirm whether SKI and ROS were able to reduce ER stress-induced apoptosis in renal tubular cells, we tested the protein expression levels of Bax, Bcl-2, and caspase12, in NRK-52E cells exposed to HG with or without SKI or ROS for 24 h. Our data indicated that the increased protein expression levels of Bax, caspase12, and GRP78, and the reduced protein expression levels of Bcl-2, in NRK-52E cells exposed to HG were significantly ameliorated following treatment with SKI or ROS. Consequently, we considered that both SKI and ROS are able to alleviate ER stress-induced apoptosis, both *in vivo* and *in vitro*.

Previous research demonstrated that activation of the PERK-eIF2α-ATF4-CHOP signaling pathway is involved in ER stress-induced podocyte apoptosis in diabetic rats, and that astragaloside IV, the main active component of SKI, can inhibit ER stress-induced podocyte apoptosis by suppressing the PERKATF4-CHOP pathway, at least in part (Chen et al., 2014). In addition, Wang et al. reported that activation of PERK-eIF2α-ATF4-CHOP signaling axis triggered by excessive ER stress leads to apoptosis in the proximal tubular cells of a rat model of nephrotoxicity (Wang et al., 2019). By regulating the activation of the PERK-eIF2α-ATF4-CHOP signaling pathway, we hypothesized that it might be possible to identify novel therapeutic mechanisms for SKI and ROS in terms of the inhibition of ER stress-induced apoptosis. Our data clearly demonstrated increased protein expression levels of p-PERK, p-eIF2α, ATF4, and CHOP in the kidneys of DKD rats and in NRK-52E cells exposed to HG. Collectively, these *in vivo* and *in vitro* findings, provided strong evidence to support the fact that the PERK-eIF2α-ATF4-CHOP signaling pathway is activated in diabetic kidneys and PTECs. Furthermore, our *in vivo* and *in vitro* experiments showed that SKI and ROS both reduced the level of phosphorylation on key signaling molecules within this key pathway, as well as the expression levels of Bcl-2 protein downstream. In addition, we found that 4-PBA (a specific inhibitor of ER stress) exerted significant suppressive effects on the activation of the PERK-eIF2α-ATF4-CHOP signaling pathway and ER stress-induced apoptosis in PTECs exposed to HG. It was clear that the *in vitro* regulatory effects of SKI on the activation of the PERK-eIF2α-ATF4-CHOP signaling pathway were similar to those evoked by a specific ER stress inhibitor. We therefore considered that SKI and ROS could suppress the activation of the PERK-eIF2α-ATF4-CHOP signaling pathway, both *in vivo* and *in vitro*.

Finally, it is important that we discuss four additional points. First, we chose 5 g/kg/day as a dose for SKI because our previous studies showed that this particular dose of SKI not only attenuated tubulointerstitial damage in the rat model of UUO model but also had no negative effects on liver function (Liu et al., 2019). Second, why we used ROS as a control in this study? Several reports show the effects of thiazolidinediones (TZDs) including ROS on ER stress. It is well known that ER stress is involved in the development of insulin resistance and type 2 diabetes mellitus, and TZDs are antidiabetic agents that act as a potent insulin sensitizer (Kang et al., 2013; Kang et al., 2020). Thus, targeting ER stress in diabetic kidneys, ROS was chosen as a positive control in this study. Third, it is important that we identify the specific targets of SKI that possess the ability to alleviate DT. Although SKI inhibited the activation of the PERK-eIF2α-ATF4-CHOP signaling pathway *in vivo* and *in vitro*, in a similar manner as 4-PBA (a specific inhibitor of ER stress), we are unable to identify the specific molecules involved in this signaling pathway. Future research should aim to identify the therapeutic targets of SKI and therefore demonstrate how the PERK-eIF2α-ATF4-CHOP signaling pathway can regulate ER stress-induced apoptosis.

## Conclusion

**FIGURE 14:**
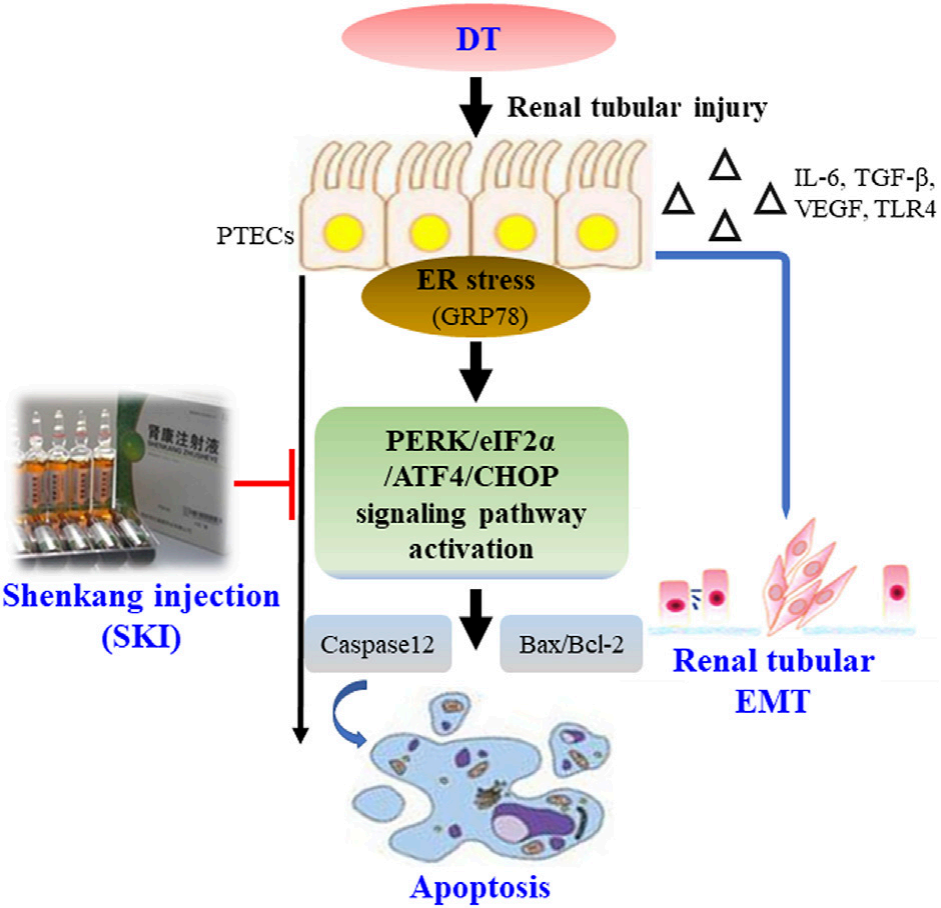
Overview of the effect of SKI on DT via the inhibition of renal tubular EMT and ER stress-induced apoptosis. Abbreviations: DT, diabetic tubulopathy; EMT, epithelial-to-mesenchymal transition; ER, endoplasmic reticulum; SKI, Shenkang injection.

This study demonstrated that SKI can alleviate DT by inhibiting EMT and ER stress-induced apoptosis, both *in vivo* and *in vitro* (Figure 14); the actions of SKI were similar to those of ROS. Our findings provide novel information relating to the potential clinical value of SKI in the treatment of DT.

## Data Availability

The original contributions presented in the study are included in the article/Supplementary Material, further inquiries can be directed to the corresponding authors.

## Supplementary Material

Supplementary Figure S1 Fingerprint analysis of SKI by HPLC. **(A)** Chromatograms produced from mixed standards, **(B)** SKI samples. Abbreviations: SKI, Shenkang injection; HPLC, high performance liquid chromatography.

Supplementary Figure S2 Animal experimental protocols.

Supplementary Figure S3 Cell viability in cultured NRK-52E cells. **(A)** Cultured NRK-52E cells were exposed to HG with SKI at 10, 20, and 40 μg/ml for 24 h; **(B)** Cultured NRK-52E cells were exposed to HG with ROS at 50, 100, and 200 nmol/L for 24 h. Abbreviations: HG, high glucose; ROS, rosiglitazone; SKI, Shenkang injection.

## Glossary

Alb: serum albumin
ATF4: activating transcription factor 4
BG: blood glucose
BUN: blood urea nitrogen
BW: body weight
collagen I: collagen type I
CHOP: C/EBP homologous protein
DKD: diabetic kidney disease
DM: diabetes mellitus;
DT: diabetic tubulopathy
eIF2α: double-stranded RNA (PKR)-activated protein kinase-like eukaryotic translation initiation factor 2α
ECM: extracellular matrix
EMT: epithelial-to-mesenchymal transition
ER: endoplasmic reticulum
GAPDH: glyceraldehyde-3-phosphate dehydrogenase
GRP78: glucose-regulated protein 78
HPLC: high performance liquid chromatography
HG: high glucose
IHC: immunohistochemistry;
IL-6: interleukin-6
KW: kidney weight
LSD: least significant difference
PAS: Periodic acid-Schiff
PERK: double-stranded RNA (PKR)-activated protein kinase-like eukaryotic translation initiation factor 2α (eIF2α) kinase
PTECs: proximal tubular epithelial cells
ROS: rosiglitazone;
SKI: Shenkang injection
Scr: serum creatinine
SDS: sodium dodecyl sulfate
STZ: streptozocin
TGF-β: transforming growth factor-β
TLR4: till-like receptor 4
TUNEL: terminal deoxynucleotidyl transferase dUTP nick end labeling
UACR: urinary microalbumin to creatinine
UNAG: urinary *N*-acetyl-beta-*d*-glucosaminidase
VEGF: vascular endothelial growth factor
WB: Western blot
α-SMA: α-smooth muscle actin
4-PBA: 4-phenylbutyric acid.
