# Supplementary figures and images for: Inhibition of Renal Tubular Epithelial Mesenchymal Transition and Endoplasmic Reticulum Stress-Induced Apoptosis with Shenkang Injection Attenuates Diabetic Tubulopathy
Source: Front Pharmacol. 2021 Aug 2;12:662706. doi: 10.3389/fphar.2021.662706 (PMC8367077; doi:10.3389/fphar.2021.662706)

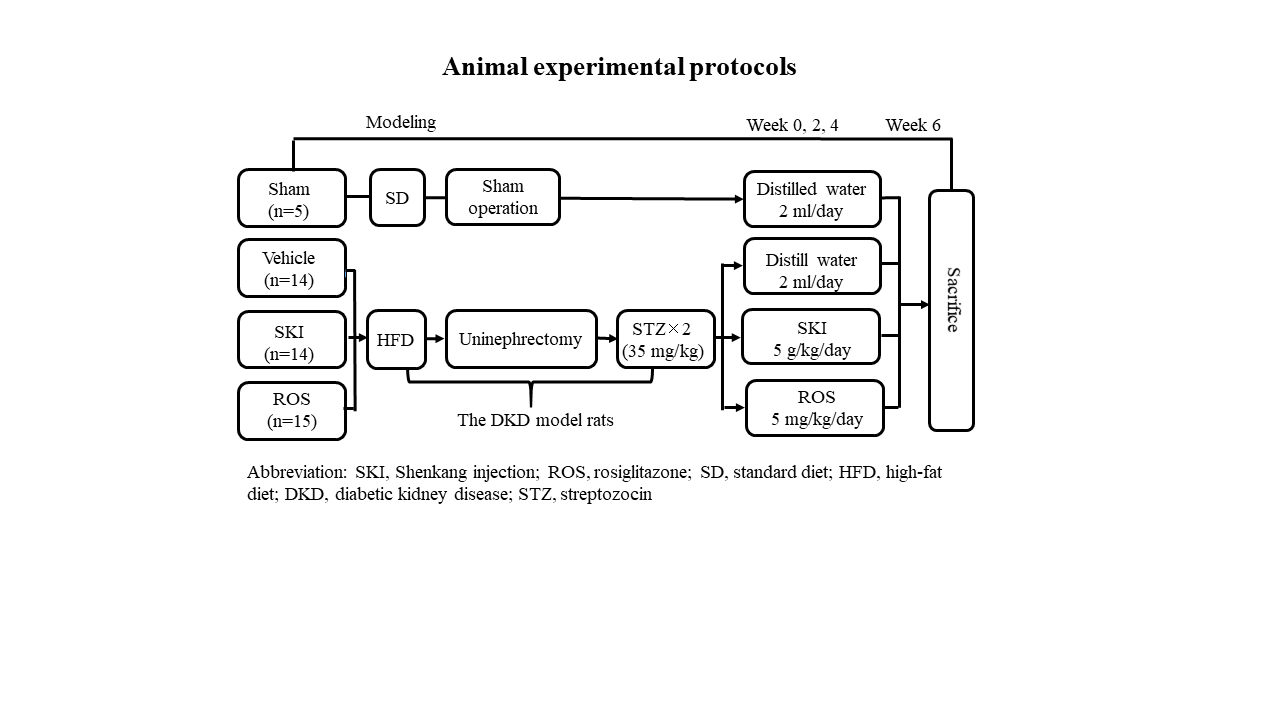

Supplement: Supplementary file 2 [file Image2.TIF]

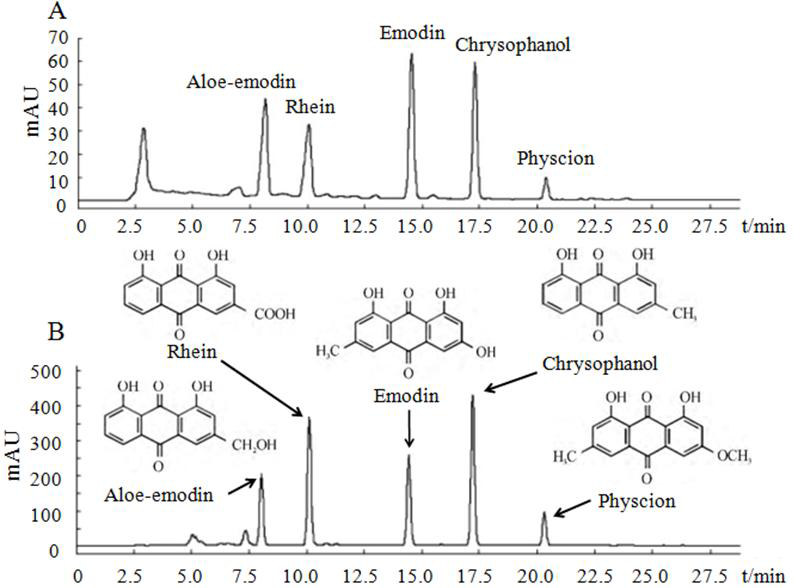

Supplement: Supplementary file 3 [file Image1.TIF]
